# Pharyngocutaneous fistula after total laryngectomy: Prevalence and risk factors

**DOI:** 10.1371/journal.pone.0348792

**Published:** 2026-06-02

**Authors:** Petronella Pettersson, Rasmus Blomkvist, Krzysztof Piersiala, Elin Marsk, Alexandra Elliot, Björn Palmgren, Claes Mercke, Linda Marklund, Rusana Bark

**Affiliations:** 1 Division of ENT Diseases, Department of Clinical Sciences, Intervention and Technology, Karolinska Institutet, Stockholm, Sweden; 2 Department of Otorhinolaryngology, Karolinska University Hospital, Stockholm, Sweden; 3 Medical Unit Head Neck, Lung and Skin Cancer, Karolinska University Hospital, Stockholm, Sweden; 4 Department of Oncology-Pathology, Karolinska Institutet, Stockholm, Sweden; 5 Department of Surgical Sciences, Section of Otolaryngology and Head and Neck Surgery, Uppsala University, Uppsala, Sweden; University of California, Davis, UNITED STATES OF AMERICA

## Abstract

**Objective:**

To determine the prevalence and identify associated risk factors of pharyngocutaneous fistula following total laryngectomy.

**Methods:**

Medical records of 160 patients diagnosed with laryngeal cancer between 2000–2021, subsequently undergoing total laryngectomy at Karolinska University Hospital, were analyzed for demographics, comorbidities, tumor characteristics, treatments and postoperative outcomes. Uni- and multivariate analyses were used to identify risk factors for pharyngocutaneous fistula.

**Results:**

Pharyngocutaneous fistula developed in 28 patients (17.5%). Univariate analysis identified cardiovascular disease (OR 2.50; 95% CI 1.00–6.28), preoperative hemoglobin <110 g/L (OR 5.34; 95% CI 1.74–16.45), prior radiotherapy (OR 3.78; 95% CI 1.24–11.52), preoperative tracheostomy (OR 2.44; 95% CI 1.05–5.72), pharyngectomy (OR 8.47; 95% CI 2.46–29.17), neck dissection (OR 2.52; 95% CI 1.06–6.00), pectoral flap reconstruction (OR 10.83; 95% CI 1.88–62.49) and postoperative infection (OR 28.44; 95% CI 9.00–89.81) as significant risk factors. In multivariate analysis only pharyngectomy (OR 7.18; 95% CI 1.08–47.63; p = 0.041) and postoperative infection (OR 24.94; 95% CI 6.66–93.46; p < 0.001) remained independent.

**Conclusions:**

Pharyngocutaneous fistula is a common complication occurring in 17.5% of patients following total laryngectomy. Pharyngectomy and postoperative infection are independent risk factors for fistula formation.

## Introduction

Laryngeal cancer, a malignancy affecting the vocal cords and/or its surrounding tissues, is one of the most common head and neck tumors with approximately 180 new cases yearly in Sweden and over 180 000 new cases worldwide [1,2]. The survival rate in small tumors is generally good, but much poorer outcomes are seen in advanced stages (T2-T4) and in presence of neck metastases (N+) [1,3–5]. In Sweden, T1N0 tumors are typically treated with a single modality, mainly surgery. Moderately advanced tumors (T2-T3, N0-N+) are generally treated with radiotherapy (RT) or chemoradiotherapy (CRT). For advanced-stage disease (T4) the primary treatment is often total laryngectomy (TL), i.e., removal of the larynx and upper trachea, combined with RT/CRT. In some cases, an additional pharyngectomy is needed, resulting in a total pharyngolaryngectomy (TPL) [6,7]. Despite curative intent with primary RT/CRT for T2-T3 tumors a significant proportion of patients develop residual or recurrent disease requiring secondary treatment with TL or TPL, referred to as salvage laryngectomy [8,9].

Patients who have undergone total laryngectomy are at risk of developing a pharyngocutaneous fistula (PCF), meaning a tract that drains saliva from the neopharynx to the skin or trachea, during the healing process. PCF is associated with significant morbidity including poor wound healing, prolonged hospitalization, delayed resumption of oral intake and in rare cases life-threatening conditions such as carotid artery rupture and mediastinitis [10–12]. Management of PCF ranges from conservative approaches like wound care to more extensive surgical procedures for fistula closure [10,12]. Although PCF is one of the most frequent complications of TL the reported incidence varies between 3–65% [13].

Previously cited risk factors for PCF include prior RT/CRT, low preoperative albumin or hemoglobin levels, comorbidities such as diabetes and cardiovascular disease, preoperative tracheostomy, concurrent neck dissection and salvage surgery [5,12–14]. The prevalence of PFC, associated risk factors, and its impact on postoperative outcomes and interventions have not been previously studied in a Nordic healthcare setting, where the majority of patients who receive RT are treated with modern IMRT techniques.

In this study we aimed to investigate the prevalence of PCF and to identify associated risk factors following TL or TPL in our patient population. We also evaluate the clinical outcomes associated with PCF. The findings may have important clinical implications for refining preoperative patient selection, guiding postoperative care, and suggesting preventive strategies for PCF.

## Materials and methods

### Study design and patient population

All patients diagnosed with laryngeal cancer in the Stockholm region and treated at Karolinska University Hospital between 1^st^ of January 2000 and 31^st^ of December 2021, who subsequently underwent treatment with TL or TPL, were included in the study.

### Data collection

Medical records were assessed during the period 09.06.2024–30.06.2024 for data collection including: age, gender, smoking, comorbidities, tumor site, TNM 7 stage, laboratory values, RT/CRT, and postoperative parameters including length of hospital stay, time to resumption of oral intake and PCF management. Studied factors are shown in detail in Tables 1–3. All data were de-identified prior to analysis.

**Table 1:** Cohort characteristics.

Parameter	Number of patients (n)	Percentage (%)
All	160	100
Gender		
*Male*	142	89
*Female*	18	11
Smoking	149	93
Diabetes	18	11
Cardiovascular disease	30	19
Hypertension	37	23
**Tumor characteristics**		
Supraglottic	40	25
Glottic	115	72
Subglottic	5	3
T1	19	12
T2	47	29
T3	40	25
T4a	54	34
T4b	0	0
N0	133	83
N+	27	17
**Treatment**		
Primary vs Salvage		
*Primary*	56	35
*Salvage*	104	65
Surgical extent		
*Laryngectomy (TL)*	148	93
*Laryngofaryngectomy (TPL)*	12	8
Neck dissection		
*No*	122	76
*Yes*	38	24
*Ipsilateral*	24	15
*Bilateral*	14	9
RT/CRT	105	66
*RT*	89	56
*CRT*	16	10
	**Mean ± SD**	**Median, range**
Age at surgery	65 ± 9.6	65, 42-89
BMI	23 ± 4.3	23, 13-35

**Table 2:** Univariate analysis of patient related factors.

Variable	All Patients n (%)	Non-fistula group n (%)	Fistula group n (%)	P -value	OR; 95% CI (p)
**Age** (mean 65 ± 9.6)					
≤ 65	84 (52.5)	67 (50.8)	17 (60.7)	0.338*	Ref.
> 65	76 (47.5)	65 (49.2)	11 (39.3)		0.667; 0.290–1.532 (0.340)
**Gender**					
Female	18 (11.3)	14 (10.6)	4 (14.3)	0.524^**†**^	Ref.
Male	142 (88.8)	118 (89.4)	24 (85.7)		0.712; 0.216–2.351 (0.577)
**Smoking**					
No	11 (6.9)	9 (6.8)	2 (7.1)	1.000^**†**^	Ref.
Yes	149 (93.1)	123 (93.2)	26 (92.9)		0.951; 0.194–4.662 (0.951)
**Diabetes**					
No	142 (88.8)	118 (89.4)	24 (85.7)	0.524^**†**^	Ref.
Yes	18 (11.3)	14 (10.6)	4 (14.3)		1.405; 0.425–4.639 (0.577)
**Astma/COPD**					
No	126 (78.8)	106 (80.3)	20 (71.4)	0.297*	Ref.
Yes	34 (21.3)	26 (19.7)	8 (28.6)		1.631; 0.646–4.114 (0.300)
**Hypertension**					
No	123 (76.9)	101 (76.5)	22 (78.6)	0.815*	Ref.
Yes	37 (23.1)	31 (23.5)	6 (21.4)		0.889; 0.331–2.387 (0.815)
**CVD**					
No	130 (81.3)	111 (84.1)	19 (67.9)	0.046*	Ref.
Yes	30 (18.8)	21 (15.9)	9 (32.1)		**2.504; 0.998–6.283 (0.051)**
**Preop Hb < 110 g/L**					
No	135 (90.0)	116 (93.5)	19 (73.1)	**0.005** ^**†**^	Ref.
Yes	15 (10.0)	8 (6.5)	7 (26.9)		**5.342; 1.735–16.445 (0.003)**
Missing	10				
**Preop albumin <32 g/L**					
No	107 (72.8)	90 (73.8)	17 (68.0)	0.555*	Ref.
Yes	40 (27.2)	32 (26.2)	8 (32.0)		1.324; 0.521–3.362 (0.556)
Missing	13				

* Chi2, ^†^ Fisher’s test, ^‡^ COPD = chronic obstructive pulmonary disease, CVD = cardiovascular disease, Preop = preoperative, Hb = hemoglobin.

**Table 3:** Univariate analysis of treatment related and postoperative factors.

Variable	All Patients n (%)	Non-fistula group n (%)	Fistula group n (%)	P -value	OR; 95% CI (p)
**Prior RT/CRT**					
No	55 (34.4)	51 (38.6)	4 (14.3)	0.014*	Ref.
Yes	105 (65.6)	81 (61.4)	24 (85.7)		**3.778; 1.239–11.520 (0.019)**
**Time RT/CRT to surgery**					
≤ 8 months	53 (50.5)	42 (51.9)	11 (45.8)	0.697*	Ref.
> 8 months	52 (49.5)	39 (48.1)	13 (54.2)		1.194; 0.487–2.927 (0.698)
**RT vs CRT**					
RT only	89 (84.8)	70 (86.4)	19 (79.2)	0.517^**†**^	Ref.
CRT	16 (15.2)	11 (13.6)	5 (20.8)		1.675; 0.518–5.409 (0.389)
**Tracheostomy**					
No	117 (73.1)	101 (76.5)	16 (57.1)	0.036*	Ref.
Yes	43 (26.9)	31 (23.5)	12 (42.9)		**2.444; 1.045–5.716 (0.039)**
**Primary vs Salvage**					
Primary	56 (35.0)	52 (39.4)	4 (14.3)		Ref.
Salvage	104 (65.0)	80 (60.6)	24 (85.7)	0.011*	**3.900; 1.279–11.888 (0.017)**
**Pharyngectomy**					
No	148 (92.5)	127 (96.2)	21 (75.0)	**0.001** ^**†**^	Ref.
Yes	12 (7.5)	5 (3.8)	7 (25.0)		**8.467; 2.457–29.171 (<0.001)**
**Neck dissection**					
No	122 (76.3)	105 (79.5)	17 (60.7)	0.033*	Ref.
Yes	38 (23.8)	27 (20.5)	11 (39.3)		**2.516; 1.056–5.996 (0.037)**
**Neck dissection**					
No	122 (77.7)	105 (79.5)	17 (60.7)	0.073*	Ref.
Ipsilateral	21 (13.4)	18 (13.6)	6 (21.4)		2.059; 0.716–5.922 (0.180)
Bilateral	14 (8.9)	9 (6.8)	5 (17.9)		**3.431; 1.026–11.477 (0.045)**
**Pectoral flap**					
No	154 (96.3)	130 (98.5)	24 (85.7)	**0.009** ^**†**^	Ref.
Yes	6 (3.8)	2 (1.5)	4 (14.3)		**10.833; 1.878–62.488 (0.008)**
**Pharyngeal suture rows**					
2	56 (39.2)	44 (37.9)	12 (44.4)	0.532*	Ref.
3	87 (60.8)	72 (62.1)	15 (55.6)		0.764; 0.328–1.781 (0.533)
Unknown	29				
**Postop Hb < 110**					
No	94 (61.8)	82 (65.1)	12 (46.2)	0.070*	Ref.
Yes	58 (38.2)	44 (34.9)	14 (53.8)		2.174; 0.926–5.105 (0.075)
**Postop infection**					
No	113 (70.6)	109 (82.6)	4 (14.3)	<0.001*	Ref.
Yes	47 (29.4)	23 (17.4)	24 (85.7)		**28.435; 9.003–89.810 (<0.001)**

* Chi2, ^†^ Fisher’s test, ^‡^ RT = radiotherapy, CRT = chemo radiotherapy, Preop = preoperative, Postop = postoperative, Hb = hemoglobin.

### Definitions

PCF was confirmed by clinical examination or radiographic imaging. Preoperative tracheostomy was defined as a tracheotomy performed on a separate occasion prior to laryngectomy for airway management. Postoperative infection was defined as clinical evidence of wound infection (such as redness, swelling, purulent discharge, foul smell etc.) requiring initiation or modification of antibiotic treatment within four weeks following surgery, in absence of salivary leakage. PCF, in contrast, was defined as salivary leakage confirmed either clinically or radiologically. Low hemoglobin was defined as hemoglobin level <110 g/L.

### Postoperative care at Karolinska University Hospital

At Karolinska University Hospital the standard postoperative care following laryngectomy includes the admission of prophylactic antibiotics, most commonly a cephalosporine in combination with metronidazole, initiated preoperatively and continued until drains are removed. A nasogastric feeding tube is placed, with an average restraint of oral intake of 10–13 days. In patients receiving a voice prosthesis, voice rehabilitation is usually initiated on the same day or the day after resumption of oral intake.

### Statistical methods

Descriptive statistics were presented as counts and percentages (n, %) for categorical variables, and as means with standard deviations (SD) or medians with ranges for continuous variables, depending on distribution. Associations between categorical predictors and the occurrence of pharyngocutaneous fistula (PCF) were evaluated using Pearson’s Chi-square test when expected cell counts were ≥5, and Fisher’s exact test when expected counts were <5. Laboratory variables were dichotomized based on institutional reference ranges. The 8-month cutoff was pre-specified as the median interval between (chemo)radiotherapy and surgery in our cohort to create two comparably sized groups and facilitate interpretability, while avoiding data-driven cutoff selection.

To assess the relative contribution of potential predictors to fistula formation, univariate logistic regression was initially performed. Variables were considered for multivariable analysis if associated with fistula formation in univariable analysis (p < 0.01), to reduce overfitting given the limited number of events. We also fitted an alternative multivariable model including prior RT/CRT despite its non-significant univariable p-value, given its established clinical relevance. An inclusion of RT/CRT did not materially change the effect estimates for the main predictors, why we retained the model without RT/CRT as the primary model for reporting.

All statistical analyses were conducted using SPSS software (version 29.0.1.0; IBM Corp., Armonk, NY, USA). A two-tailed p-value ≤ 0.05 was considered statistically significant.

### Ethics

This study was approved by The Swedish Ethical Review Authority (Ethical no: 2019–04829 with the amendment Ethical no: 2021-06907-02 and Ethical no: 2023-05249-01 with the amendment 2025-03148-02). Data were retrieved from medical records in compliance with ethical permissions above. In accordance with the approval of the Regional Ethics Committee, the requirement for informed consent was waived, as the study involved only retrospective analyses.

## Results

### Patient characteristics

A total of 757 patients were diagnosed with laryngeal cancer at Karolinska University Hospital between the 1^st^ of January 2000 and 31^st^ of December 2021. Out of these, 160 were identified to have gone through TL or TPL. The majority were male (89%) and the mean age was 65 years (range 42–89). Most patients (93%) had a history of smoking. The most common primary tumor subsite was in the glottic region (72%). Patient characteristics are summarized in Table 1.

The median follow-up time was 37 months (range 1–220 months). A total of 105 patients (65,6%) had previously received RT/CRT, while 55 patients (34.4%) had not undergone prior RT/CRT. A majority of the patients received IMRT, which was introduced at our institution on April 1, 2005. Of the 105 patients treated with radiotherapy, 36 were treated prior to this date, although all treatments during the study period utilized highly conformal 3D-techniques. Primary surgical treatment with TL or TPL was performed in 56 patients (35%), while 104 patients (65%) underwent salvage surgery. All patients selected for primary surgery had T4a tumor stage. The tumor stage in the salvage cases ranged from T1 to T3 at diagnosis with a median time of 8 months (range 0–97 months) from completion of primary RT/CRT to salvage surgery. Regardless of whether the surgery was primary or salvage, all but 14 patients received a voice prosthesis during the same procedure as the laryngectomy.

### Prevalence of pharyngocutaneous fistula

Out of the 160 patients included in the study, 28 (17.5%) developed a pharyngocutaneous fistula (PCF). The mean time from surgery to diagnosis of PCF was 18 days (range 5–37).

### Risk factors for PCF formation

In a univariate analysis of patient-related factors potentially associated with PCF formation (Table 2), we found that cardiovascular disease (OR 2.50; 95% CI 1.00–6.28; p = 0.046) and low preoperative hemoglobin at a level of less than 110 g/L (OR 5.34; 95% CI 1.74–16.45; p = 0.005) were significantly associated with increased risk of PCF formation. Other patient factors, including age, gender, smoking status, diabetes, pulmonary disease, and hypertension, were not significantly associated with PCF formation in our cohort.

In the univariate analysis of treatment-related factors (Table 3), we observed a significantly increased risk of postoperative PCF in patients who had undergone prior radiotherapy (OR 3.78; 95% CI 1.24–11.52; p = 0.019). All but one of these patients had received RT as a primary curative treatment, while the remaining patient received it as neoadjuvant therapy. Time from RT to surgery, defined as either shorter or longer than the mean of 8 months, did not affect PCF risk. Neither did the addition of chemotherapy to the prior radiotherapy.

Patients who underwent preoperative tracheotomy had a 2.4-fold increased risk of developing PCF (OR 2.44; 95% CI 1.05–5.72; p = 0.039) compared to those who did not undergo preoperative tracheotomy. A greater extent of surgical resection, with pharyngectomy in addition to total laryngectomy, was also associated with increased risk of PCF formation (OR 8.47; 95% CI 2.46–29.17; p < 0.001), as was concurrent neck dissection (OR 2.52; 95% CI 1.06–6.00; p = 0.037). When stratifying by the extent of neck dissection, bilateral neck dissection was significantly associated with PCF formation (OR 3.43; 95% CI 1.03–11.48; p = 0.045), while ipsilateral neck dissection only showed a non-significant trend toward increased risk (OR 2.06; 95% CI 0.72–5.92; p = 0.180). Six patients underwent reconstruction with a pectoral flap, which posed a 10.8 times greater risk of PCF formation (OR 10.83; 95% CI 1.88–62.49; p = 0.008). The number of suture rows (2 vs. 3) applied in pharyngeal closure did not affect the risk of PCF in our cohort.

Analysis of postoperative factors (Table 3) revealed that postoperative infection greatly increased the incidence of PCF, with a 28.4 -fold higher risk of PCF formation (OR 28.44; 95% CI 9.00–89.81; p < 0.001) compared to cases not complicated by infection. No perioperative optimization of hemoglobin or albumin was performed, and no standardized albumin supplementation or blood transfusions were used. Postoperative hemoglobin levels, however, did not have an impact on the risk of PCF formation.

### Multivariate analysis of PCF formation

In the multivariate analysis, which included variables with a p-value of <0.01 in univariate analysis and prior RT/CRT, only pharyngectomy (OR 7.18; 95% CI 1.08–47.63; p = 0.041) and postoperative infection (OR 24.94; 95% CI 6.66–93.46; p < 0.001) remained independent predictors of postoperative PCF development (Table 4). Notably, preoperative low hemoglobin levels and pectoral flap reconstruction did not retain statistical significance in the multivariate model.

**Table 4:** Multivariate analysis of risk factors for PCF formation.

Variables with p < 0.01 in univariate analysis	P-value	aOR; 95% CI
**Pharyngectomy**		
No	**0.039**	Ref.
Yes		**8.067; 1.111-58.566**
**Pectoral flap**		
No	0.341	Ref.
Yes		3.222; 0.289–35.862
**Preoperative Hb < 110 g/L**		
No	0.434	Ref.
Yes		1.833; 0.402-8.346
**Postoperative infection**		
No	**<0.001**	Ref.
Yes		**27.594; 7.477-101.840**

### Clinical outcomes related to PCF

Among the 28 patients who developed a postoperative PCF, the duration of hospital stay was 60% longer compared to the non-fistula group, with a mean stay of 32 versus 20 days, respectively. All patients with PCF were treated with antibiotics, either initiated due to the PCF or modified if the PCF developed during an ongoing course of prophylactic antibiotics. An additional 39% of PCF patients received treatment in a hyperbaric oxygen chamber, all of whom were treated between 2000–2011. Surgical intervention was performed in 36% of PCF cases. The median time to recovery of oral intake was 68 days in the fistula group, compared to 13 days in the non-fistula group. In patients with a voice prosthesis who developed PCF, the mean time to initiation of voice use after the PCF diagnosis was 84 days, compared to the standard of approximately 14 days postoperatively in our clinic. Of the 28 patients with PCF, four had a persistent fistula – defined as one that had not resolved by the time of death or at the patient’s last follow up. One patient died due to a carotid bleed associated with the PCF.

## Discussion

Our observed rate of PCF of 17.5% is consistent with previous reports in the literature [13]. Pharyngectomy and postoperative infection were identified as independent risk factors while cardiovascular disease, preoperative hemoglobin <110 g/L, prior RT/CRT, tracheostomy, salvage surgery, neck dissection and the use of a pectoral flap only were significant in the univariate analysis. Furthermore, we observed similar findings regarding the impact of PCF on patient outcomes, with PCF resulting in prolonged hospitalization, delayed resumption of oral intake, and postponed voice rehabilitation [15–17]. Given the frequency of PCF occurrence and its associated morbidity, continued investigation of this complication remains critically important, especially since the remaining heterogeneity regarding risk factors presents a challenge in establishing consistent predictors and preventive measures for PCF formation [14].

Pharyngectomy emerged as an independent risk factor in the multivariate analysis, aligning with previous findings that more extensive resections involving the pharynx increase the risk of wound complications [11,18]. The larger defect in the pharynx likely creates greater tension along the suture line in the neopharynx, which, when combined with other factors such as tissue compromised by prior radiotherapy, increases the risk of rupture and fistula formation. For patients requiring pharyngectomy, a thorough evaluation of risk factors, preoperative optimization, and consideration of reinforcement with vascularized tissue for reconstruction—particularly in irradiated cases—are especially important.

Postoperative infection was identified as the strongest independent risk factor for PCF in our study. This relationship may reflect a bidirectional association, as infections can lead to fistula formation, and fistulas can precipitate infection [19]. However, our clinical observations suggest that signs of infection often precede fistula development, which emphasizes the importance of meticulous wound care by experienced medical personnel who can promptly identify early signs of infection and initiate appropriate treatment.

Prior radiotherapy, often overlapping with salvage surgery, is frequently reported as a risk factor for PCF and was associated with fistula formation in our univariate analysis [20–23]. However, it did not persist as an independent predictor in multivariate analysis. Although RT/CRT is well known from earlier studies to negatively affect microcirculation and tissue healing, our findings suggest that its impact may be less pronounced in this setting. This could potentially reflect the transition to more sparing IMRT techniques. Another possible explanation is the relationship between previous radiotherapy and other factors in our study, particularly postoperative infection, which was strongly associated with PCF. Radiation-damaged tissue, with compromised vascularity, may predispose patients to both infection and fistula formation, making it difficult to distinguish these factors statistically [10]. A majority of patients in the present cohort underwent salvage total laryngectomy. Since prior (chemo)radiotherapy and compromised tissue vascularity are more common in salvage settings, the observed associations in the overall cohort should be interpreted primarily in the context of salvage-heavy case mixes. Predictors of PCF may differ between primary and salvage laryngectomy; however, the limited number of PCF events among primary procedures in this material precludes a robust subgroup analysis without substantial risk of spurious findings. Consequently, our estimates represent overall associations and may not be directly generalizable to cohorts dominated by primary laryngectomy.

Preoperative hemoglobin levels below 110 g/L were significantly associated with PCF in univariate analysis but not in multivariate analysis. Low hemoglobin has been associated with impaired wound healing in various surgical procedures and specifically to PCF in multiple studies [12,18]. Our findings may suggest that anemia may contribute to PCF development, although its effect becomes less pronounced when accounting for other factors. Regardless, preoperative optimization of anemia appears to be a reasonable intervention, particularly in patients with multiple risk factors.

Cardiovascular comorbidity approached statistical significance as a risk factor for PCF, consistent with previous studies showing that conditions affecting microcirculation can impair wound healing [11,24]. Although cardiovascular disease did not emerge as an independent risk factor in our analysis, it remains a relevant consideration in the comprehensive risk assessment of patients undergoing total laryngectomy.

Preoperative tracheostomy has been identified as a potential risk factor for PCF in several studies, with proposed mechanisms related either to tumor size or to tissue contamination during the procedure [12,15,25]. Our findings in univariate analysis support this potential association.

The use of pectoralis major flap reconstruction has shown varying impact on the occurrence of PCF in the literature, with some studies suggesting a protective effect through the provision of well-vascularized, non-irradiated tissue, while others have found an increased risk of PCF [26]. Gil et al. demonstrated that prophylactic flap placement reduced PCF incidence by 42% in high-risk patients [27], while Guimarães et al. found an association with increased PCF risk, likely reflecting selection bias for more complex cases requiring reconstruction [28]. In our cohort, although univariate analysis showed that pectoral flap increased the risk for PCF, only a small number of patients underwent pectoralis flap reconstruction. This suggests that these cases were more advanced, in which a higher risk of PCF was expected.

Our finding of neck dissection being associated with increased PCF rates in the univariate analysis is consistent with several previous studies [11,25,29]. This emphasizes the importance of further investigation on diagnostic methods for lymph node management, which could potentially minimize the extent of neck dissection and associated morbidity. For example, sentinel lymph node biopsy has shown promising potential, with high detection rates and sensitivity for neck metastasis [30,31].

Beyond identifying risk factors for PCF development, the presence of established PCF and its impact on oncologic outcomes, cancer recurrence, and patient survival is of substantial clinical importance and has been addressed in recent research. In a multicenter retrospective collaborative study, the development of PCF after salvage laryngectomy was associated with more than a two-fold increased risk of distant metastasis [32]. Studies investigating the relationship between local inflammation and malignant transformation have further focused on mechanisms involving immune suppression and the generation of tumor cells with an increased propensity for metastasis [33]. Whether these observations are supported by the results of the present study has not been specifically examined, as this was considered beyond the primary scope of the study, which focused on identifying predictive factors for the development of PCF.

Our study has several strengths, including a relatively large single-center and contemporary Nordic cohort, which likely ensures a relatively homogeneous approach to laryngectomy procedures, program-regulated RT/CRT with respect to volume, delivered doses and fractionation, and postoperative care. Such consistency strengthens the internal validity of the findings consistent with our experience in managing these patients. However, several limitations should be acknowledged. The retrospective study design and the relatively small number of patients who developed PCF limit the statistical power and may have reduced our ability to detect some associations in both univariate and multivariate analysis. Accordingly, the results should be interpreted with appropriate caution. Furthermore, evidence in the literature suggests a possible association between surgical margin status and PCF [34]. In the present study, surgical margin status was not included in the analysis due to incomplete data from the early period and the consequent risk of introducing selection bias.

## Conclusion

PCF remains a common complication following TL/TPL, posing significant challenges for both patients and healthcare providers. In our study, we found that patients requiring an additional pharyngectomy or those who develop postoperative infections are at greater risk for PCF formation. Patients with risk factors for developing PCF warrant particular attention before, during and after surgery. Future prospective studies with larger patient cohorts are needed to better understand the complex interplay of factors contributing to PCF development and to establish evidence-based prevention strategies.
